# The Role of Bcl-xL Protein Research in Veterinary Oncology

**DOI:** 10.3390/ijms21072511

**Published:** 2020-04-04

**Authors:** Aleksandra Pawlak, Marta Henklewska

**Affiliations:** Department of Pharmacology and Toxicology, Faculty of Veterinary Medicine, Wroclaw University of Environmental and Life Sciences, 50-375 Wrocław, Poland; marta.henklewska@upwr.edu.pl

**Keywords:** Bcl-xL, comparative oncology, canine cancers, apoptosis

## Abstract

Due to their significant impact on human and animal health, cancer diseases are an area of considerable concern for both human and veterinary medicine. Research on the cancer pathogenesis in companion animals, such as dogs, allows not only for improving canine cancer treatment, but also for translating the results into human oncology. Disruption of apoptosis in tumor-transformed cells is a well-known mechanism leading to the development of cancer. One of the main factors involved in this process are proteins belonging to the B-cell lymphoma 2 (Bcl-2) family, and the imbalance between pro-apoptotic and anti-apoptotic members of this family contributes to the development of cancer. Studies on the function of these proteins, including B-cell lymphoma-extra large (Bcl-xL), have also been intensively conducted in companion animals. The Bcl-xL gene was sequenced and found to share over 99% homology with the human protein. Research showed that the Bcl-2 family plays the same role in human and canine cells, and data from studies in dogs are fully translatable to other species, including humans. The role of this protein family in cancer development was also confirmed. The article presents the current state of knowledge on the importance of the Bcl-xL protein in veterinary oncology.

## 1. Introduction

### 1.1. Comparative Oncology—Canine Models in Cancer Biology and Therapy

Due to the similarity between dog and human cancers, the importance of comparative oncology has increased significantly in recent times. It is a rapidly developing discipline aimed at integrating studies on naturally occurring animal cancers and employing these data in a more general research on cancer biology and therapy [1]. This type of research includes the study of cancer pathogenesis as well as the search for new therapeutic options for different types of cancer [2]. Of various animal species that develop cancer (e.g., cat, horse, rabbit, ferret), a dog turned out to be the most appropriate model due to its body size, life expectancy, cancer incidence, and sharing of living conditions with humans [3,4]. Moreover, dogs together with mice and rats are popular laboratory animals necessary for different types of toxicological studies [5].

The problem of cancer in dogs poses a considerable challenge to modern veterinary medicine. This can be evidenced by the fact that in the United States, more than a million dogs are diagnosed with cancer every year, and retrospective studies clearly show that cancer has become the leading cause of dog deaths with an estimated rate of ∼30% [6]. This requires treating canine patients but at the same time offers a possibility of conducting clinical trials of new therapeutic options. There are many advantages of such a model and the most important of them include the following: (a) spontaneous occurrence of tumors in immunocompetent individuals; (b) naturally developing treatment resistance of the cancer cells; (c) spontaneous recurrence and metastasis; (d) possibility of canine patients monitoring for hematologic and biochemical toxicity via routine clinical pathology, and sophisticated monitoring like in human medicine; (e) ability to notice the impact of supportive care (e.g., antiemetics, antidiarrheals, antibiotics, etc.) on the course of a therapy; (f) occurrence of heterogeneity within the cancer [7]. All of the above advantages encourage scientists to design research involving dogs and the results obtained this way become a valuable source of medical information. Moreover, natural consequences of the mentioned heterogeneity are the most deadly features of all cancers and thus companion animal cancers capture the “essence” of the contemporary oncology problem. No other animal model system allows for observation of the phenomenon of tumor heterogeneity [1].

The most important types of cancer that can be studied using canine disease models include, e.g., bone tumors, such as osteosarcoma, hematopoietic cancers—especially leukemia and non-Hodgkin’s lymphoma, melanoma, various carcinomas of the lung, bladder, head and neck, prostate or mammary gland, soft tissue sarcomas, and multiple brain cancer types [7]. These types of cancer, in addition to similar genetic background and clinical course in dogs and humans, also share a similar clinical approach. In veterinary medicine, the most important element of a therapy is a surgical removal of neoplastic lesions and it is still the major therapeutic method used in all types of solid tumors, such as bone and mammary gland tumors. It is also the basis for the treatment of soft tissue sarcomas and different types of carcinoma, melanoma, or mastocytoma [1]. With the increasing availability of advanced medical devices, cancer therapy in animals is assisted by radiation therapy. Chemotherapy is also used as an adjuvant treatment in the aforementioned types of cancer in the form of traditional cytostatic drugs of high effectiveness in specific cancer cells. Commonly used therapeutic protocols are based on the drugs widely used in human medicine: anthracyclines, platinum derivatives, or vinca alkaloids. In metastatic and hematopoietic cancers, chemotherapy remains the main therapeutic method. Therapeutic programs used in these types of cancer are also extrapolated from human medicine and include typical human protocols containing glucocorticosteroids, alkylating agents, anthracyclines, antimetabolites, and vinca alkaloids. For some types of cancer, e.g., mastocytomas, molecularly targeted drugs—tyrosine kinase inhibitors—are available in veterinary medicine. Currently, two small molecule inhibitors, toceranib and masitinib, have been registered in veterinary medicine for clinical use [6]. There is already a wide spectrum of different types of cancers sharing similarities in dogs and humans, and further research is likely to increase the pool that may be of interest to comparative oncology. In 2005, the results of the Canine Genome Project were published in which 99% of the dog’s genome was sequenced. All of the approximately 19,000 genes showed similarity to human genes [8]. Moreover, most if not all of the cancer-associated genetic alterations that determine cancer progression in humans were identified in the canine cancers [9,10,11,12,13,14,15,16]. This clearly proved that canine and human genomes are similar enough to suggest that information obtained in one species can be transferred to and can be applicable in the other. One of the topics in which canine models can be used are studies on apoptosis and the possibility of its induction in the cancer cells. The Bcl-xL (B-cell lymphoma-extra large) protein is the subject of the presented research and one of the most important components of the apoptotic process.

### 1.2. Role of Bcl-xL Protein in Apoptosis

Cell turnover (programmed cell death—apoptosis) is essential for organism development during embryogenesis. Apoptosis is also crucial for the elimination of infected, damaged, or pathologically changed cells [17]. Furthermore, the normal function of the intestinal epithelium, mammary glands, as well as reproductive and immune systems depends on the active, programmed cell death [18]. The factors promoting cell survival (e.g., growth factors, cytokines) or factors triggering cell death regulate these processes by downstream pathways and different families of proteins. Some of the most important players regulating programmed cell death belong to the family of pro- and anti-apoptotic proteins named B cell lymphoma 2 (Bcl-2). The proteins belonging to this family contain at least one of Bcl-2 homology (BH) domains that account for the interactions between Bcl-2 proteins crucial for controlling mitochondrial outer membrane permeability (MOMP) [17]. The members of the Bcl-2 family are divided into three groups. The division is based on the number of Bcl-2 homology domains and the function of individual proteins. The three groups are a) "BH-3-only" pro-apoptotic Bcl-2 proteins, e.g., BIM (Bcl-2 interacting mediator of cell death), BAD (Bcl-2-antagonist of cell death), BID (BH-3 interacting domain death agonist), (p53 upregulated modulator of apoptosis), NOXA, and many others; b) multi-domain pro-apoptotic executioner proteins BAX (Bcl-2 associated X ), BAK (Bcl-2 antagonist/killer), and BOK (Bcl-2 related ovarian killer), and c) multi-domain pro-survival proteins inhibiting BH-3-only and executioner proteins, such as Bcl-2, Bcl-xL, Bcl-w, Mcl-1 (myeloid cell leukemia 1), and A1 [18,19,20].

Bcl-2 family is mainly involved in the mitochondrial (intrinsic) apoptotic pathway triggered by various cytotoxic stresses (stimuli), such as genotoxic agents, intracellular damage, or cytokine deprivation. Under normal conditions in healthy cells the apoptosis is prevented by a balance between pro- and anti-apoptotic members of the Bcl-2 family and their physical interactions [21]. Pro-survival proteins bind to both pro-apoptotic BH-3-only and multi-domain proteins blocking their pro-apoptotic activity. Under stress conditions, BH-3-only Bcl-2 members are induced transcriptionally or post-translationally, which leads to their quantitative advantage over the anti-apoptotic proteins and initiates apoptosis. The process is triggered by directly activating the pro-apoptotic Bcl-2 proteins (by BH-3-only proteins called activators) or by neutralizing the anti-apoptotic Bcl-2 proteins that cause indirect activation of pro-apoptotic members of Bcl-2 the family (by BH-3-only proteins called sensitizers) [18]. 

An essential step in initiating the apoptosis is the activation of BAX–BAK monomers by BH-3-only proteins. More detailed studies demonstrated that important mechanisms of the regulation of this process involve an interaction between the anti-apoptotic Bcl-2 family members and BH-3-only activators and sensitizers. Bcl-xL and Bcl-2 prevent the apoptosis by sequestering the activator BH-3s from BAX–BAK, while after an apoptotic stimuli BH-3-only sensitizers enable interactions between the multi-domain pro-apoptotic executioner proteins and their activators by displacing the anti-apoptotic proteins from the activators [22,23]. The anti-apoptotic effects of Bcl-xL protein (as well as Bcl-2 or Mcl-1) can be prevented by BID, BIM, and PUMA proteins while NOXA can only inhibit Mcl-1. It is worth remembering that the remaining BH-3s, including BAD, BMF (Bcl-2 modifying factor), BIK (Bcl-2-interacting killer), and HRK (harakiri, Bcl-2 interacting protein) cannot activate BAX–BAK directly and appear to promote the apoptosis only by preventing Bcl-2 and Bcl-xL from sequestering activator BH-3s [22]. In addition to the presented mechanism, some factors involved in the induction of apoptosis are known to trans-activate the multi-domain pro-apoptotic Bcl-2 proteins, as can be shown on the example of tumor suppressor p53 and BAX protein [24]. 

As a result of a direct activation by BH-3-only activators, BAX and BAK oligomerize and form mitochondrial permeability transition pores (MPT) in the outer mitochondrial membrane [17]. The mitochondrial outer membrane permeabilization enables a release of pro-apoptotic factors, such as cytochrome C, Smac/DIABLO (second mitochondria derived activator of caspases/direct inhibitor of apoptosis-binding protein with a low isoelectric point), and serine protease HtrA2 (high temperature requirement protein A2)/Omi. Cytochrome C interacts with an adaptor protein apoptotic protease activating factor 1 (APAF-1) forming an apoptosome which in turn promotes activation of caspase 9. Smac/DIABLO facilitates caspase cascade by inhibiting anti-apoptotic caspase inhibitory proteins called IAPs (inhibitor of apoptosis proteins) [25]. The apoptosome formed by the released cytochrome C, active caspase 9, and APAF-1 is essential for activating the effector caspases 3, 6, and 7. These caspases are responsible for cleaving various proteins in the cell causing morphological and biochemical changes associated with an executioner phase of the apoptosis [26].

## 2. Bcl-xL Protein and Its Canine Version

### Structure and Function of Bcl-xL Protein in Apoptosis

Isolation of a human *Bcl-x* gene was first reported by Boise et al. in 1993, and the gene was found to encode two protein products, Bcl-xL and Bcl-xS [27]. Subsequently, the structure of the proteins was determined enabling a deeper understanding of the interactions between the pro- and anti-apoptotic Bcl-2 family members. The structure of the Bcl-xL protein consists of eight α-helices (1–8), forming, like in other anti-apoptotic Bcl-2 family members, four BH domains and a C-terminal hydrophobic region responsible for anchoring to the membranes (C-terminal transmembrane domain). The BH-1 domain is located on the turn region linking the helices α4 to α5, and the BH-2 domain can be found on the turn region between the helices α7 to α8. The BH-3 domain is located on helix α2, while the BH-4 domain is located on α1. An important element of the protein structure is a hydrophobic groove formed by the domains 1–3, which is the site of the interaction with the BH-3 domain of pro-apoptotic proteins [28].

The coding sequence of the canine *Bcl-xL* gene was also successfully cloned and sequenced. The studies of Sano et al. (2003) revealed that a full length of canine cDNA clone contained 1252 bp and a single reading frame of 699 bp encoding a protein of 233 amino acids [29]. Most importantly, it was shown to share over 99% homology with the human protein. A high (over 97%) level of homology was also found for mouse, rat, sheep, and pig, which confirmed the results of earlier studies [27], indicating that the Bcl-xL gene is highly conserved among the species. Moreover, the structural organization of the canine Bcl-xL with four domains was the same as in other species [29]. In more recent studies, de Brot et al. (2016) confirmed that the coding sequence of the canine Bcl-xL gene contains 702 bp and is distributed over two exons [30]. For comparison, the feline Bcl-xL gene was also sequenced by Sano et al. (2005) and was found to share 99.1% and 98.7% sequence homology with the dog and human counterparts, respectively. The protein organization was identical to the Bcl-xL of the other species [31].

Under normal conditions, the physiological balance between the pro- and anti-apoptotic members of the Bcl-2 family and their impact on the cell fate depends on various factors affecting the cell and acting through intracellular signal transduction pathways. The presence of the anti-apoptotic members of the Bcl-2 family and their contribution to the apoptosis inhibition is part of the system ensuring the cell survival. The involvement of Bcl-xL in the cell survival and inhibition of apoptosis results from the positive regulation of its transcription by factors engaged in the cell survival. For example, JAK/STAT (Janus kinase/Signal transducer and activator of transcription) and PI 3-kinase signaling pathways support the survival of hematopoietic progenitor cells in response to cytokine and growth factors mainly by regulating Bcl-xL gene expression [32,33,34]. It is not surprising that a high Bcl-xL expression, among other factors involved in cell proliferation, cellular transformation, and preventing apoptosis, has been linked to the constitutive activation of STAT proteins in many cancers and associated with the apoptosis resistance [35]. Furthermore, a nuclear factor kappaB (NF-kB), responsible for modulating expression of the proteins involved in the immune and inflammatory response [36], is capable of directly activating Bcl-xL [37,38,39]. On the other hand, in response to pro-apoptotic stimuli the factors engaged in the pro-apoptotic signaling can participate in the process by inactivating the Bcl-xL protein. It was shown, for example, that following DNA damage, the stress-activated protein kinase/c-Jun NH_2_-terminal kinase (SAPK/JNK) translocated to the mitochondria, bound to Bcl-xL protein, and phosphorylated Bcl-xL on threonine 47 (Thr-47) and threonine 115 (Thr-115), which may have inhibited its anti-apoptotic activity [40].

As mentioned earlier, the anti-apoptotic members of the Bcl-2 family prevent apoptosis by restraining BAX–BAK monomers from homo-oligomerization and binding to the pro-apoptotic BH-3-only and multi-domain Bcl-2 family members. Bcl-xL can directly interact and form complexes with BAX [18,41], BAK [42], and possibly BOK [28], and sequester the activator BH-3-only proteins [23,43] by forming complexes with BIM [44,45], BID [45], and PUMA [44]. Due to its high stability and dual inhibition of BAX and BAK, Bcl-xL is considered more efficient than Bcl-2 and Mcl-1 in preventing DNA damage-induced apoptosis [46].

In addition, Bcl-xL contributes to the prevention of apoptosis through an additional mechanism involving sequestration of the active cytosolic form of p53. Stress stimuli, such as DNA damage, leads to p53 protein stabilization and its accumulation in the nucleus and cytosol. While the nuclear p53 is responsible for triggering the transcription of the pro-apoptotic factors, its cytosolic form was shown to directly activate BAX [47]. Bcl-xL is capable of directly binding and sequestering cytosolic p53 protein to prevent direct activation of the pro-apoptotic proteins by cytosolic p53 [48]. This mechanism can account for additional prevention of apoptosis in the case of a too weak apoptotic signal, since PUMA-dependent release of cytosolic p53 from its inactive complexes with Bcl-xL is necessary for increasing the strength of the apoptotic signal [49]. On the other hand, p53 was shown to release the pro-apoptotic proteins from their inhibitory complexes with anti-apoptotic members of the Bcl-2 family, including Bcl-xL [47]. Bcl-xL can directly interact with caspases. Interestingly, this interaction serves two opposite functions, i.e., probable inhibition of caspase activity and cleavage of Bcl-xL in the loop region by caspase 1 and 3 lead to a release of a C-terminal fragment with the pro-apoptotic activity, which in turn can serve as a mechanism amplifying the pro-apoptotic signal [50]. In addition to its well established role in the mitochondrial apoptotic pathway, Bcl-xL is capable of preventing the apoptosis and supporting cell survival by regulating Ca^2+^ homeostasis, releasing Ca^2+^ from the endoplasmic reticulum (ER) (Ca^2+^ channels in ER) [51,52,53] and limiting mitochondrial Ca^2+^ uptake, thus protecting from Ca^2+^ overload and Ca^2+^ mediated apoptosis [54].

## 3. Bcl-xL in Canine Cancer Studies

**Figure 1:**
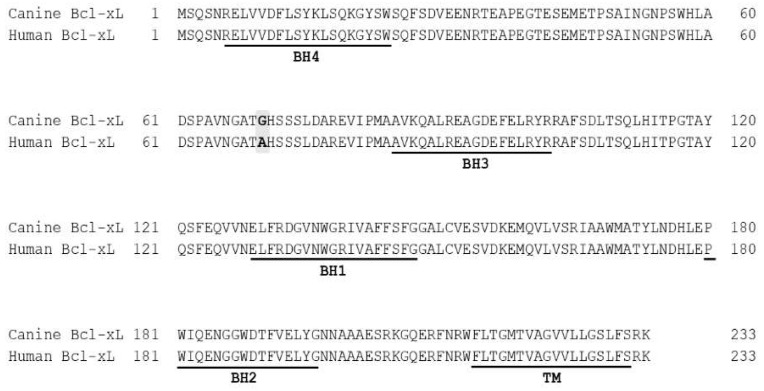
Amino acids sequences alignment of canine and human Bcl-xL after Sano J 2003. The sequences of Bcl-2 homology domains (BH 1-4) and transmembrane domain (TM) are underlined. Variant residues are highlighted in bold and gray background.

Research on the possibility of apoptosis induction in canine cancer cells and the mechanisms responsible for such an action are an increasingly frequent topic of investigation in the contemporary veterinary oncology. The structure and function of the individual components of the apoptotic pathway are also intensively studied. The paper by Sano et al. published in 2002 was crucial to understanding the structure and function of Bcl-xL protein in canine cancer cells (Figure 1) [29]. 

The structure of the canine Bcl-xL based on the information from this article and an article by de Brot et al. (2016) [30] was discussed in detail in the previous paragraph. It should be noted, however, that the results of studies on the function and interactions between the canine anti-apoptotic proteins, such as Bcl-xL, Bcl-w, and Mcl-1, and the pro-apoptotic proteins like BAX and BAK, indicate a significant similarity in the role and mode of action of the proteins from the Bcl-2 family in dogs and other species, including humans [30]. These results confirm the hypothesis on usefulness of the canine cells as a research model in the comparative oncology. In the mentioned paper of Sano, the authors cloned and sequenced for the first time the canine Bcl-xL gene, and examined the expression of Bcl-xL mRNA using semi-quantitative RT-PCR (reverse-transcription polymerase chain reaction) in different canine cancer cell lines. Their study showed that mRNA for the tested protein was constitutively expressed in two canine lymphoma (CL-1) and leukemia cell lines (GL-1) as demonstrated by a comparison with the expression level in PBMC (peripheral blood mononuclear cells) obtained from healthy donors. This suggests that survival and/or resistance to the apoptosis observed in the canine hematopoietic cancers may be associated with changes in the expression of Bcl-xL protein. The results of the above-mentioned study encouraged other researchers to look closer at the changes in the expression of Bcl-xL protein in various types of cancers, and the aforementioned lymphoma and leukemia cell lines CL-1 and GL-1 became a model for in vitro development of new cancer therapies based on the induction of apoptosis in tumor-transformed canine cells.

The expression of the Bcl-xL protein was studied in canine neuroepithelial tumors. Of all domestic animals this type of cancer most often affects dogs and unfortunately it is still incurable [55]. The search for new therapeutic targets, including the possibility of inducing apoptosis in these cancer cells is therefore an important scientific and clinical problem. Immunohistochemical characterization showed an intense positive response to Bcl-2 and Bcl-xL in choroid plexus tumors, primitive neuroectodermal tumors (PNETs), and neuroblastomas. In astrocytomas, a significantly higher percentage of cells expressing Bcl-xL protein compared to Bcl-2 protein was found, whereas a reverse trend was observed in oligodendrogliomas [56]. High expression of the anti-apoptotic proteins from the Bcl-2 family may therefore be the cause of cell resistance to apoptosis in these types of cancers. Most likely, the balance between the pro- and anti-apoptotic proteins is shifted here in favor of those molecules that block the process of apoptosis. Clinically, such a situation in the cells can manifest itself in rapid development of the tumor and its resistance to treatment. In the observed type of tumors, the inhibitors of the anti-apoptotic proteins from the Bcl-2 family (pan-Bcl-2 inhibitors) seemed to be a useful therapeutic option that requires investigation. In contrast to choroid plexus tumors, PNETs, neuroblastomas, astrocytomas, and oligodendrogliomas gliomatosis cerebri were almost negative for both Bcl-2 and Bcl-xL [56]. Therefore, other cancer therapies should be considered in these particular types of cancers.

The importance of imbalance between the pro- and anti-apoptotic proteins from the Bcl-2 family was also studied in mammary gland tumors in an in vitro model using a canine CF33 cell line (ATCC CRL6227). Tsuchiya et al. (2006) not only showed that Bcl-xL can be a therapeutic target in canine mammary gland cancers, but also that mRNA expression of this protein can be silenced in canine cells by using appropriate siRNA [57]. In this study, the authors presented the effect of mRNA silencing (using oligonucleotides in Oligofectamine) of Bcl-xL on the course of apoptosis in CF33 cells. The experiment demonstrated a clear increase in the percentage of apoptotic cells following their 48 h incubation with siRNA (small interfering RNA) for Bcl-xL. TUNEL (Tdt-mediated dUTP nick-end labeling) method confirmed that after downregulation of Bcl-xL, the level of apoptosis in CF33 cell lines was 60.9% vs. 28.7% in control (cells incubated with oligofectamine medium only). This result indicated that the Bcl-xL protein is one of the factors blocking apoptosis of the mammary gland tumor cells and perhaps this protein should be the target of new therapies for this type of cancer in dogs too. At the same time, this study described experimental conditions that allow gene silencing in the canine cells and showed considerable cytotoxicity of oligofectamine medium itself (induction of apoptosis in about 30% of cells). This high toxicity certainly limits the possibility of long-term gene silencing using the described method. The field was further explored by Nagamatsu et al. (2008) who used another, less toxic method (transfection with siRNA and cationic liposomes) to investigate the effect of Bcl-2 gene silencing on the pro-apoptotic activity of doxorubicin on CF33 (canine mammary carcinoma) cells [58]. Although the study concerned another anti-apoptotic protein, Bcl-2, it showed that the imbalance between the pro- and anti-apoptotic proteins may significantly increase sensitivity of the examined cancer cells to the cytostatic drugs commonly used in the therapy. It also indicated new therapeutic options in the canine mammary tumors where silencing or blocking of the function of Bcl-2 protein could be a method of increasing cell sensitivity to doxorubicin. It should be expected that a similar relationship can be demonstrated for Bcl-xL protein.

Another type of cancer in which the expression and significance of Bcl-2 family proteins, including Bcl-xL, was studied was mastocytoma—a very common type of cancer in dogs. Importantly, this disease in dogs and humans bears a significant similarity [59]. In both species, the disease is characterized by mutations in c-kit as well as a resistance to classical cytostatic drugs. As cancerous mast cells (MC) in aggressive systemic mastocytosis (ASM) and mast cell leukemia (MCL) were found to express the anti-apoptotic Mcl-1, Bcl-2, and Bcl-xL, Peter et al. (2014) decided to examine the effects of obatoclax (GX015-070), the pan-Bcl-2 family blocker on human and canine mastocytoma cell lines [60]. The study showed that obatoclax-mediated inhibition of the proteins from the Bcl-2 family effectively curbed proliferation of the cancerous mast cells and induced their apoptosis. Moreover, viral-mediated overexpression of Mcl-1, Bcl-xL, or Bcl-2 in HMC-1 cells induced partial resistance to the apoptosis-triggering effects of obatoclax. Additionally, this in vitro study demonstrated synergistic effects of obatoclax and some tyrosine kinase inhibitors, such as midostaurin, dasatinib, and bortezomib, effectively inducing the apoptosis or inhibiting the growth of cancerous mast cells. Thus, mastocytoma seems to be another type of cancer in which new therapeutic directions may be associated with targeting the proteins that block apoptosis. As this type of cancer often occurs in dogs, this potentially new therapy can be first introduced in dogs and provide valuable observations before starting human clinical trials.

Based on the reports on the role of Bcl-xL protein in resistance to cytostatic drugs, research into new compounds with potential anti-tumor activity, involving canine cells as a model, also focused on the effects the new compounds may have on the expression and/or function of the Bcl-xL protein. Such a group of chemicals are, for example, aromatic lactones, both of natural origin and their synthetic derivatives, whose biological activity is currently being intensively studied [61,62,63,64,65,66,67]. Our studies on iodolactones employed cell lines overexpressing the anti-apoptotic proteins. This allowed us to select the compounds that affected Bcl-2 protein expression levels. This strategy proved effective as we managed to identify the compounds capable of inducing apoptosis despite an initial advantage of the anti-apoptotic over pro-apoptotic proteins. It was demonstrated that the mechanism of action of the tested enantiomeric trans b-aryl-d-iodo-c-lactones results from the induction of classical caspase-dependent apoptosis as a result of imbalance between the pro- and anti-apoptotic proteins. The study clearly showed a decrease in Bcl-xL and Bcl-2 protein expression in canine lymphoma cell lines under the action of both tested compounds, however, it was demonstrated that (+)-(4R, 5S, 6R)-1 isomer was characterized by higher activity [68]. Taken together, these findings, despite slight differences in individual cell lines, indicated that the enantiomeric trans b-aryl-d-iodo-c-lactones derived from 2,5-dimethylbenzaldehyde act through inducing a decrease in Bcl-2 and Bcl-xL levels, followed by caspase activation and PARP cleavage. Therefore there is a possibility that the investigated compounds might be used in adjuvant treatment that would sensitize the cancer cells to apoptosis.

Another important study in the veterinary experimental oncology on the expression and function of the Bcl-xL protein was published by Henklewska et al. in 2019 [69]. The authors examined the anticancer properties of a complex of platinum with tris(2-carboxyethyl)phosphine (Pt-TCEP), a novel platinum derivative. The study was performed using a model of the most common bone tumor types in humans and dogs—osteosarcoma U2-OS and D-17 cell lines. It showed that Bcl-xL protein expression declined after exposure to 5 μM Pt-TCEP for 24 hours. These results are consistent with the previous reports, according to which downregulation of Bcl-xL expression leads to apoptosis of cisplatin treated cells [70]. Additionally, the D-17 cell line used in the study overexpressed Bcl-2 and Bcl-xL proteins. Overexpression of proteins from the Bcl-2 family in the canine osteosarcoma cell line further confirmed the importance of research on these proteins in veterinary oncology. A higher basal expression of the anti-apoptotic Bcl-2 and Bcl-xL proteins in D-17 than in U2-OS cell line may account for lower sensitivity to Pt-TCEP of the canine osteosarcoma cell line. It is in fact one of the primary mechanisms of cancer chemoresistance, also in the case of platinum-based drugs [71]. Perhaps bone tumors in dogs are another type of cancer in which higher rates of therapy efficacy could be obtained after using Bcl-2 family inhibitors. Further research is needed to determine the frequency of overexpression of these proteins in bone cancer in dogs.

Although canine cells and canine neoplasms are a recognized model for investigating certain types of human cancers, sometimes observations from this model provide different results than expected based on the findings of mouse or human research. An example of a study using canine cancer cells that did not confirm previous observations on the effects of the Bcl-xL protein are our experiments on the possibility of sensitizing lymphoma cells to TRAIL ligand by using flavopiridol, a cyclin-dependent kinase inhibitor [72]. Numerous literature reports indicate that a combination of TRAIL (TNF-related apoptosis inducing ligand) and flavopiridol limits the expression of the anti-apoptotic proteins from the Bcl-2 family (including Bcl-xL), wherein the pro-apoptotic proteins gain advantage and apoptosis is induced in the cells treated with this combination [73,74,75,76]. Surprisingly, these studies in canine lymphoma cells reported no changes in the expression levels of XIAP, Mcl-1, Bcl-2, and Bcl-xL proteins following treatment with either flavopiridol alone or with the combination of flavopiridol and human TRAIL. However, a clear reduction of an anti-apoptotic cFLIP (FADD-like IL-1β-converting enzyme) was observed after treating the cells with the mentioned combination of drugs. This indicates that apoptosis inhibition due to overexpression of the anti-apoptotic proteins is also a reason for the resistance to TRAIL ligand, but the Bcl-xL protein is at least not the main cause of the observed resistance in canine lymphoma cells.

Research on Bcl-xL in veterinary oncology is not limited to dogs. Dogs are the most numerous but not the only group of patients in the oncological veterinary clinics, which is why scientific research also applies to other animal species, for example, cats. In one recent study, expression of Bcl-2 and Bcl-xL in feline lymphoma cell line FT-1 was evaluated upon in vitro treatment with common anticancer drugs [31]. The authors also sequenced a full-length feline Bcl-xL gene and showed its high homology with the predicted Bcl-xL of dog, human, mouse, pig, rat, and sheep. The levels of Bcl-2 transcripts after 24 h incubation of FT-1 cells stimulated with doxorubicin (0.3 µg/mL), prednisolone (0.2 µg/mL), and vincristine (5 ng/mL) increased by about 41.0, 62.0, and 11.1 times as compared with the non-stimulated FT-1 cells. The level of Bcl-xL transcripts after 24 h incubation of FT-1 cells stimulated by doxorubicin and prednisolone increased by about 4.2 and 5.8 times vs. control, and the inducible level of Bcl-xL decreased by about 0.35 times in the presence of vincristine [31]. The enhanced expression of the anti-apoptotic proteins after the use of cytostatics is undoubtedly a negative result from the perspective of therapeutic expectations and may be the reason for the observed resistance to the therapy. Faced with this observation, it seems reasonable to include Bcl-2 blockers in standard therapeutic protocols to allow the drug-damaged cells to enter the apoptosis pathway. However, another study using feline lymphoma cell lines and feline lymphocytes to investigate the expression of proteins involved in the apoptosis during cell infection with FIV (feline immunodeficiency virus), showed no changes in Bcl-xL protein expression [31]. In this case, FIV infection did not affect the levels of BAX, Bcl-xL, and caspase 3, measured at mRNA level, although the infection induced apoptosis in Kumi-1 cells. At the same time, FIV intensified the expression of Bcl-2 mRNA in the lymphocytes of the established cell line, which may indicate that the Bcl-2 protein is the one responsible for resistance of FIV positive cells to apoptosis.

Changes in Bcl-xL protein expression may not only be important in veterinary oncology. One study also attempted to assess changes in Bcl-2 proteins expression and their effect on the apoptosis in canine neutrophils after stimulation with lipophilysaccharide (LPS). In Sano et al.’s research from 2005, the authors indicated that Bcl-xL and BAX levels in canine neutrophils were significantly affected by LPS stimulation. They found that in neutrophils the levels of Bcl-xL, Bcl-2, Mcl-1, and BAX mRNAs after 9 h of stimulation with LPS (100 ng/mL) increased by about 80.4, 1.9, 1.4, and 5.3 times in comparison with the non-stimulated neutrophils. This means that Bcl-xL protein plays also an important role in the inhibition of canine neutrophil apoptosis in contact with LPS [77]. This type of research can provide key information on the innate immune systems of dogs, which in turn is important to prevent and combat infectious diseases, which are still, along with cancer, the leading causes of death among dogs.

## 4. Conclusions

The above outline of the state-of-the-art research in veterinary oncology clearly indicates a growing importance of studies on apoptosis, the role of Bcl-2 family proteins, and in particular Bcl-xL protein in developing new therapeutic strategies and explaining mechanisms of drug action and resistance to commonly used cytostatic drugs. The increasing number of specialized research tools available in veterinary experimental oncology makes it possible to conduct studies in canine or feline cancer cells, which are an irreplaceable model for preclinical research. The results of studies on the function and interactions between dog anti-apoptotic and pro-apoptotic proteins indicate that there is a significant similarity in the role and mode of action of proteins from the Bcl-2 family in dogs and other species, including humans. Such a statement should be the first step to integrate the studies of biochemists, biologists, clinicians, and veterinarians, because only such a comprehensive approach can ensure rapid progress in the fight against cancer. Companion animals serving as basic research models and patients prove to be invaluable allies in this fight.

## Abbreviations

APAF-1	apoptotic protease activating factor 1
ASM	aggressive systemic mastocytosis
BAD	Bcl-2-antagonist of cell death
BAK	Bcl-2 antagonist/killer
BAX	Bcl-2 associated X
Bcl-2	B-cell lymphoma 2
Bcl-xL	B-cell lymphoma-extra large
BH	Bcl-2 homology
BID	BH-3 interacting domain death agonist
BIK	Bcl-2 interacting killer
BIM	Bcl-2 interacting mediator of cell death
BMF	Bcl-2 modifying factor
BOK	Bcl-2 related ovarian killer
cFLIP	Cellular FLICE (FADD-like IL-1β-converting enzyme)-inhibitory protein
ER	endoplasmic reticulum
FIV	Feline immunodeficiency virus
HRK	harakiri, Bcl-2 interacting protein
HtrA2	high temperature requirement protein A2
IAPs	inhibitor of apoptosis proteins
JAK/STAT	Janus kinase/Signal transducer and activator of transcription
LPS	Lipopolysaccharide
MC	mast cells
MCL	mast cell leukemia
Mcl-1	myeloid cell leukemia 1
MOMP	mitochondrial outer membrane permeability
MPT	mitochondrial permeability transition
NF-kB	nuclear factor kappaB
PBMC	peripheral blood mononuclear cells
PI 3-kinase	phosphatidylinositol 3-kinase
PNETs	primitive neuroectodermal tumors
Pt-TCEP	complex of platinum with tris(2-carboxyethyl)phosphine
PUMA	p53 upregulated modulator of apoptosis
RT-PCR	reverse-transcription polymerase chain reaction
SAPK/JNK	stress-activated protein kinase/c-Jun NH_2_-terminal kinase
siRNA	small interfering RNA
Smac/DIABLO	second mitochondria derived activator of caspases/direct inhibitor of apoptosis binding protein with a low isoelectric point
TRAIL	TNF-related apoptosis inducing ligand
TUNEL	Tdt-mediated dUTP nick-end labeling
XIAP	X-linked inhibitor of apoptosis
